# Progression of cognitive and behavioural impairment in early amyotrophic lateral sclerosis

**DOI:** 10.1136/jnnp-2020-322992

**Published:** 2020-05-13

**Authors:** Emma Beeldman, Rosanne Govaarts, Marianne de Visser, Michelle Klein Twennaar, Anneke J van der Kooi, Leonard H van den Berg, Jan H Veldink, Yolande A L Pijnenburg, Rob J de Haan, Ben A Schmand, Joost Raaphorst

**Affiliations:** 1 Department of Neurology, Amsterdam University Medical Centres, Amsterdam, The Netherlands; 2 Department of Neurology, University Medical Center Utrecht, Utrecht, The Netherlands; 3 Clinical Research Unit, Amsterdam University Medical Centers, Amsterdam, The Netherlands; 4 Department of Psychology, University of Amsterdam, Amsterdam, The Netherlands

**Keywords:** ALS, cognition, behavioural disorder, frontotemporal dementia

## Introduction

Whether cognitive and behavioural impairment in amyotrophic lateral sclerosis (ALS) is progressive is unknown. The majority of longitudinal studies (summarised in online supplementary material) are characterised by stable test results, high attrition and under-representation of patients with a short disease duration.^1 2^ The aim of this study was to determine progression of cognitive and behavioural impairment in patients with early symptomatic ALS.

10.1136/jnnp-2020-322992.supp1 Supplementary data

## Methods

At our tertiary ALS clinics, we consecutively recruited patients with ALS with a short disease duration (<12 months). Comprehensive neuropsychological examination and behavioural assessment were conducted at baseline and at 6 months. Patients with behavioural variant frontotemporal dementia (bvFTD) and healthy controls (HC) served as control groups. We used validated tests to correct for motor and speech impairment, for example, the verbal fluency index. Inclusion and exclusion criteria and neuropsychological tests, including parallel versions are listed in online supplementary material 1. Test scores were considered abnormal below the fifth percentile, corrected for age and education.^3^

Patients with ALS were classified according to consensus criteria, as having ‘no’, ‘mild’ or ‘severe’ cognitive impairment. ‘No’ is determined by ≤1 abnormal neuropsychological test (letter fluency excluded), ‘mild’ by impaired letter fluency, or impairment on either two (non-overlapping) executive tests or two (non-overlapping) language tests (maximum of 3 abnormal tests); ‘severe’ by >3 abnormal tests (including letter fluency, and either two executive or two language tests, as described above).^3^

We used the ALS-FTD-Questionnaire (ALS-FTD-Q) to subdivide patients into groups with ‘no’ (<22 points), ‘mild’ (≥22 and <29 points) or ‘severe’ (≥29) behavioural impairment based on validated cut-off scores.

We further assessed *C9orf72* status, motor function (ALS Functional Rating Scale-Revised (ALSFRS-R)), respiratory function (forced vital capacity (FVC)) and anxiety and depression (Hospital Anxiety and Depression Scale (HADS)); for detailed descriptions, see online supplementary material 1.

### Statistical analysis

Change scores (follow-up minus baseline, raw scores) of neuropsychological tests and the ALS-FTD-Q were examined within groups (Wilcoxon signed rank test) and between groups (Kruskal-Wallis test; or Mann-Whitney U test, where appropriate). Shifts between categories (‘no’, ‘mild’, ‘severe’) of cognitive and behavioural impairment at baseline and follow-up were recorded for each patient. We compared change scores of the ALSFRS-R, FVC and HADS between patients with ALS with and without category shifts using Kruskal-Wallis test. The occurrence of new impairment on cognitive tests at follow-up was evaluated for individual participants.

## Results

We included 35 patients with ALS with a median disease duration of 8 months (range 4–15) and a mean age of 63.8 years (SD 8.4). Twenty-one bvFTD patients (including 5 ALS-bvFTD patients) and 18 HC were included. Groups were matched on age, education and sex (p=0.4, p=0.2 and p=0.3, respectively, online supplementary material). At baseline, nine patients with ALS (26%) had mild cognitive and/or behavioural impairment; and another seven (20%) were severely affected. The proportion of patients with bulbar onset or the presence of affective symptoms did not differ between these groups (online supplementary material).

Follow-up data were obtained at a mean of 6.1 months (SD 0.1) in 28 patients with ALS (80%), 19 bvFTD patients (91%) and 18 HC (100%). Main reasons for loss to follow-up were death, fast disease progression and fatigue.

### Group level

Change scores for letter and category fluency, verbal memory, executive function and social cognition differed between groups, with lower scores in patients with ALS and bvFTD compared with HC (online supplementary material). Change scores did not differ between patients with ALS and ALS-bvFTD.

Change scores within groups showed a decline for category fluency in patients with ALS, and for category fluency, social cognition and executive function in bvFTD patients. HC had higher scores on letter fluency and verbal memory at follow-up.

ALS-FTD-Q change scores differed between patients with ALS and HC, but not between bvFTD and HC (online supplementary material).

### Individual level: shifts between categories

**Figure 1:**
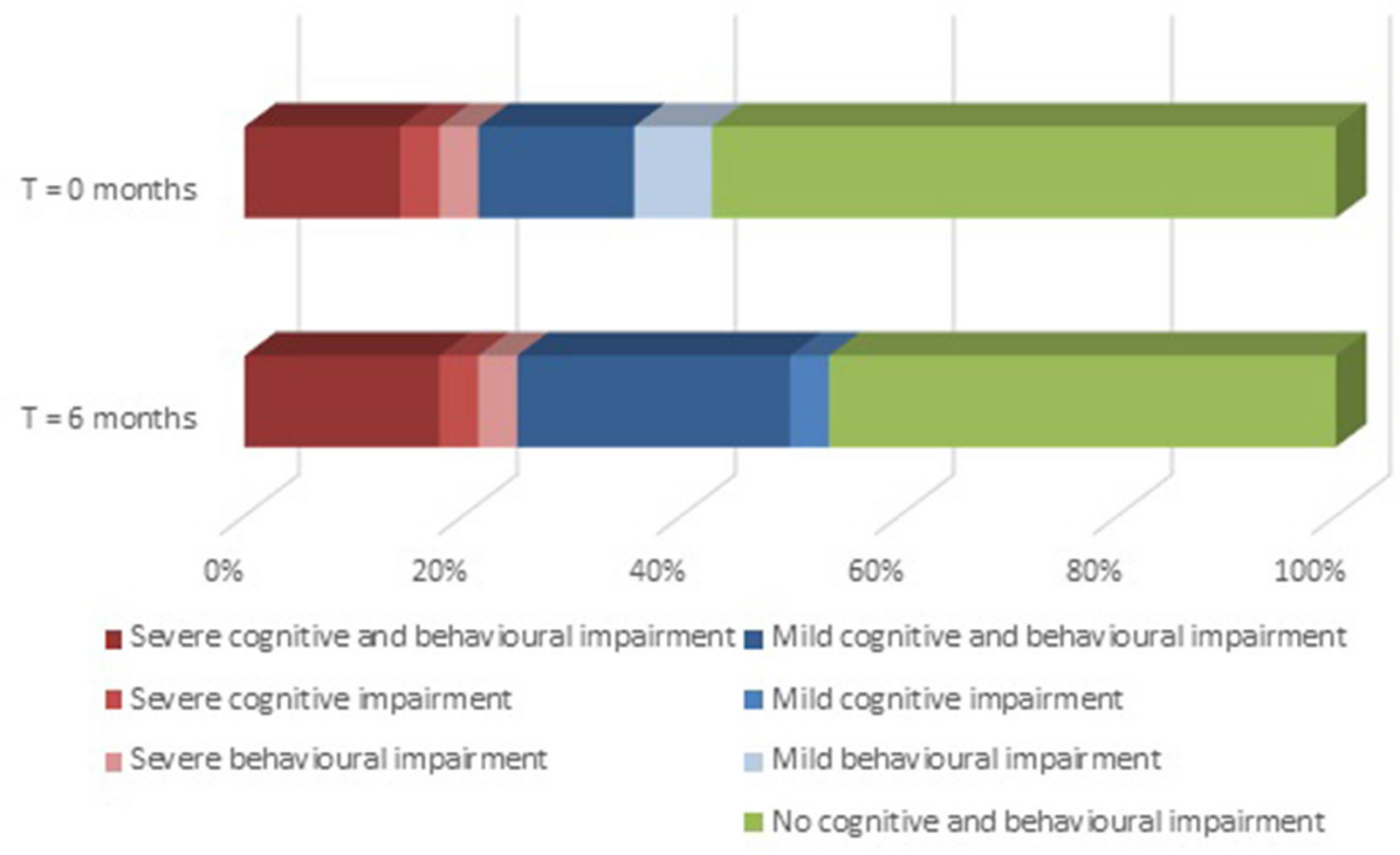
Category shifts among patients with ALS at follow-up. Proportions of patients with ALS (n=28) with no (green), mild (blue) or severe (red and pink) cognitive and/or behavioural impairment at baseline (upper bar) and follow-up (lower bar). Severe behavioural impairment is compatible with the behavioural variant of frontotemporal dementia. Ten patients shifted towards a more severe category: six from ‘no’ to ‘mild’ cognitive impairment, one from ‘no’ to ‘mild’ behavioural impairment, two from ‘no’ to ‘severe’ behavioural impairment and one from ‘no’ to ‘mild’ cognitive impairment and ‘severe’ behavioural impairment. ALS, amyotrophic lateral sclerosis.

Ten out of 28 patients with ALS (36%) shifted towards a more severe category (from ‘no’ to ‘mild’ or ‘severe’ cognitive and/or behavioural impairment), of whom one had a *C9orf72* mutation (figure 1). Fifteen patients with ALS (54%) fell into the same category at baseline and follow-up. Three patients with ALS (11%) shifted from ‘mild’ to ‘no’ cognitive and/or behavioural impairment. Change scores of ALSFRS-R, FVC and HADS did not differ between patients with and without category shifts (online supplementary material).

Cognitive impairment at the individual level was also examined by occurrence of new impairment on cognitive tests. Twelve patients with ALS (43%) without cognitive impairment at baseline, had one (n=7) or two (n=5) new abnormal tests at follow-up, mostly category fluency (n=7), antisaccade test (n=4) and Ekman 60 faces test (n=3). Four patients with ALS with executive dysfunction at baseline showed new impairment at follow-up on tests of executive, memory, language and visuoperceptive functions. The remaining 12 patients, that is, 7 without and 5 with cognitive impairment at baseline, had no new abnormal tests at follow-up.

## Discussion

The findings of a decline of social cognition in patients with ALS and declines of executive and verbal memory functions confirm that cognitive dysfunction is a widespread feature in ALS, even in early disease.^1^ It cannot be ascribed to physical, respiratory or affective deterioration, since these were comparable between cognitively stable and progressive patients. Improvement, probably a practice effect, was found in HC on most tests. A lack of practice effects in ALS may be an early sign of frontotemporal dysfunction.

Our study adds to existing literature by showing that in patients with early symptomatic ALS behavioural decline may occur, compatible with the development of bvFTD.^2^ Consequently, our study shows that the frontotemporal syndrome of ALS is not only relevant in the later stages of the disease.

In addition to its strengths (unique cohort, longitudinal design, low attrition), our study has some limitations: groups of patients were relatively small and no disease specific cognitive screening measure was used.

In conclusion, we found progression of cognitive and/or behavioural impairment in more than one third of patients with early symptomatic ALS. This study may fuel ongoing discussions about the inclusion of cognitive and behavioural symptoms in the diagnostic criteria of ALS.^4 5^
