# Supplementary material for: Progression of cognitive and behavioural impairment in early amyotrophic lateral sclerosis
Source: J Neurol Neurosurg Psychiatry. 2020 May 13;91(7):779–80. doi: 10.1136/jnnp-2020-322992 (PMC7361007; doi:10.1136/jnnp-2020-322992)
Supplement: Supplementary data [file jnnp-2020-322992supp001.pdf]

Online supplementary material

"Progression of cognitive and behavioural impairment in early amyotrophic lateral sclerosis"

Emma Beeldman MD[1*], Rosanne Govaarts MSc[1*], Marianne de Visser MD, PhD[1], Michelle Klein Twennaar MSc[1], Anneke J. van der Kooi MD, PhD[1], Leonard H. van den Berg[2], Jan H. Veldink MD, PhD[2], Yolande A.L. Pijnenburg MD, PhD[3], Rob J. de Haan PhD[4], Ben A. Schmand PhD[5], Joost Raaphorst MD, PhD[1]

Amsterdam University Medical Centers, University of Amsterdam, Department of Neurology, Amsterdam Neuroscience, Amsterdam[1], University Medical Center Utrecht, Rudolf Magnus Institute, Utrecht[2], Amsterdam University Medical Centers, Vrije Universiteit, Alzheimer Centre, Amsterdam Neuroscience, Amsterdam[3], Amsterdam University Medical Centers, University of Amsterdam, Clinical Research Unit, Amsterdam[4], Amsterdam University Medical Centers, University of Amsterdam, Department of Medical Psychology, Amsterdam[5], all in the Netherlands

Corresponding author: Emma Beeldman, Amsterdam UMC, University of Amsterdam, Department of Neurology, P.O. Box 22700 DE Amsterdam, The Netherlands. Telephone: +31-20-5663856; Fax: +31-20-5669374; E-mail: e.beeldman@amsterdamumc.nl

Beeldman E, *et al. J Neurol Neurosurg Psychiatry* 2020; 91:779–780. doi: 10.1136/jnnp-2020-322992

Contents

	Page
1. Overview of longitudinal studies investigating cognitive and/or behavioural impairment in ALS (table 1)	3
2. Selection procedure and inclusion and exclusion criteria	6
3. Neuropsychological test battery	8
4. Measures of behaviour, motor function, respiratory function, anxiety and depression	9
5. Participant and disease characteristics at baseline	10
6. Comparison of relevant participant and disease characteristics at baseline between patients with ALS with and without cognitive and/or behavioural impairment	11
7. Change scores for neuropsychological tests and behavioural assessments at follow-up	12
8. Comparison of change scores of motor function, respiratory functions and anxiety and depression between patients with ALS with and without category shifts	14
9. References	15

1. Overview of longitudinal studies investigating cognitive and/or behavioural impairment in ALS

Studies examining cognition and behaviour							
**Author, year**	**N**	**Follow-up interval (N)**	**Attrition rate**	**Disease duration baseline**	**Type of neuropsychological examination (full or screen) and behavioural screening**	**Cognition at BL<br>Cognition at FU**	**Behaviour at BL<br>Behaviour at FU**
Burkhardt, 2017(1)	40	6 mo. (n=24)<br>12-18 mo. (n=10)	40.0%<br>75.0%	44.4 mo.	Screen: ECAS, FAB<br>Behaviour: ECAS	BL: 20.8% impaired letter fluency<br>FU: no decline	BL: n/a<br>FU: no decline
Floeter, 2017(2)	15	6 mo. (n= n/a)<br>12 mo. (n= n/a)<br>18 mo. (n= n/a)	n/a	24.4 mo.	Full; domains: executive, language*<br>Behaviour: FBI	BL: n/a<br>FU: decline in letter fluency	BL: n/a<br>FU: no decline
Poletti, 2018(3)	168	6 mo. (n=48)<br>12 mo. (n=18)<br>24 mo. (n=5)	71.4%<br>89.3%<br>97.0%	19.0 mo.	Screen: ECAS, FAB, MOCA<br>Behaviour: ECAS	BL: ECAS 37% impaired<br>FU: improvement	BL: ECAS 41% impaired<br>FU: no decline
Woolley, 2018(4)	294	12 mo. (n=134)	54.4%	14.2 mo.	Full; domain executive<br>Screen: ALS-CBS<br>Behaviour: ALS-CBS, FBI-ALS	BL: ALS-CBS 60.7% impaired<br>FU: no decline	BL: ALS-CBS 30.6% impaired<br>FU: ALS-CBS 26.1% impaired. Decline on group level on ALS-CBS and FBI-ALS
Xu, 2017(5)	108	6 mo. intervals (n= n/a)	n/a	n/a	Screen: ACE-III, FAB, ECAS<br>Behaviour: MIND-B, ALS-FTD-Q	BL: ACE 30% impaired,<br>FAB 14% impaired.<br>ECAS 22% impaired<br>FU: no decline	BL: ALS-FTD-Q 32.1% impaired,<br>MIND-B 39.4% impaired<br>FU: n/a
**Studies examining cognition**							
**Author, year**	**N**	**Follow-up interval (N)**	**Attrition rate**	**Disease duration baseline**	**Type of neuropsychological examination (full or screen)**	**Cognition at BL<br>Cognition at FU**	
Abrahams, 2005(6)	20	6 mo. (n=20)	0%	20.6 mo.	Full; domains: executive, language, visuoperception	BL: impairment on fluency	

Beeldman E, et al. J Neurol Neurosurg Psychiatry 2020; 91:779–780. doi: 10.1136/jnnp-2020-322992

						FU: decline on 1 language test, improvement on 1 language test and visuoperception
Elamin, 2013(7)	186	6 mo. (n=98)<br>12 mo. (n=46)<br>18 mo. (n=11)	47.3%<br>75.3%<br>94.1%	n/a	Full; domains: executive, memory, language, visuoconstruction	BL: 49.4% impaired<br>FU: decline on visuoconstruction<br>Impaired patients at BL: decline on language, memory and visuoconstruction<br>Non-impaired patients at BL: no decline
Gillingham, 2017(8)	20	9 mo. (n=11)	45.0%	44.4 mo.	Full; domains executive, visuoperception, language<br>Screen: ALS-Computerized frontal battery; domains social, executive	BL: impairment on executive functions<br>FU: no decline
Kasper, 2016(9)	93	3-6 mo. intervals (n = n/a)	n/a	± 30 mo.	Full; domain: executive*	BL: impairment in 23.7%<br>FU: no decline
Kilani, 2004(10)	18	6 mo. (n=14)<br>12 mo. (n=13)	22.2%<br>27.8%	n/a	Full; domains: executive, language, memory	BL: impairment in executive functions and language<br>FU: no decline
Proudfoot, 2015(11)	61	6, 12, 18, 24 mo. (n=29)	50.8%	38.6 mo.	Full; domain: executive	BL: impairment on all tests<br>FU: no decline
Robinson, 2006(12)	19	6 mo. (n= n/a)	n/a	< 12 mo.	Full; domains: executive, memory, visuoperception	BL: no impairment<br>FU: decline in 36.8% on verbal memory, working memory, visual organization and visuoperception
Schreiber, 2005(13)	52	4 mo. (n=32)<br>8 mo. (n=24)<br>12 mo. (n=19)	38.5%<br>53.8%<br>63.5%	27.2 mo.	Full; domains: executive, memory, attention	BL: impairment in executive and memory<br>FU: decline in 1 executive test, 1 memory test, 1 attention test<br>Improvement in 1 executive test and 1 memory test
Strong, 1999(14)	13	6 mo. (n=8)	38.5%	21.1 mo.	Full; domains: executive, memory, visuospatial, language	BL: impairment on visuospatial<br>FU: No decline

Beeldman E, *et al. J Neurol Neurosurg Psychiatry* 2020; 91:779–780. doi: 10.1136/jnnp-2020-322992

Studies examining behaviour						
**Author, year**	**N**	**Follow-up interval (N)**	**Attrition rate**	**Disease duration baseline**	**Type of behavioural screening**	**Behaviour at BL**<br>**Behaviour at FU**
De Silva, 2016(15)	21	9-17 mo. (n=5)	76.2%	33.4 mo.	Behaviour: FTD-FRS	BL: 47.6%<br>FU: trend of decline

Legend. Overview of longitudinal studies investigating cognitive and/or behavioural impairment in ALS, arranged by type of examination (respectively cognition and behaviour, cognition only, behaviour only) and first author. BL: baseline measurement; FU: follow-up measurement; mo.: months; y: years; n/a: not available; ECAS: Edinburgh cognitive and behavioural amyotrophic lateral sclerosis (ALS) screen; FAB: frontal assessment battery; MOCA: Montreal cognitive assessment; ALS-CBS: ALS cognitive and behavioural screen; FBI-ALS: frontal behavioural inventory – ALS; FBI: frontal behavioural inventory; ACE-III: Addenbrooke's cognitive examination – III; MIND-B: motor neuron disease behaviour scale; ALS-FTD-Q: ALS – frontotemporal dementia – questionnaire; FTD-FRS: FTD – functional rating scale. *Parallel versions of the neuropsychological tests were used at follow-up.

Beeldman E, *et al. J Neurol Neurosurg Psychiatry* 2020; 91:779–780. doi: 10.1136/jnnp-2020-322992

2. Selection procedure and inclusion and exclusion criteria

We prospectively recruited patients with amyotrophic lateral sclerosis (ALS) between September 2013 and December 2016 at tertiary referral centers for ALS in The Netherlands (Amsterdam University Medical Centers and University Medical Centre Utrecht). For comparison, we recruited a group of disease controls, i.e. patients with behavioural variant frontotemporal dementia (bvFTD) or ALS-bvFTD, at a tertiary referral center for dementia (Amsterdam University Medical Centers). Healthy controls (HC) were recruited through the ALS Foundation Netherlands and participating patients (spouses, family members, friends). This study was performed in agreement with the Declaration of Helsinki. The local medical ethical committee approved the study. Written informed consent was obtained from all participants at inclusion.

Inclusion and exclusion criteria

Diagnosis in patients with ALS was probable, probable-laboratory supported or definite ALS, according to the revised El Escorial criteria.(16) Cases could be sporadic or familial (with or without a known mutation). Disease duration had to be less than 12 months in order to fulfil the criterion "early symptomatic ALS" as described previously.(17) Disease onset was defined as the time of the first ALS-related symptom (bulbar dysfunction or limb muscle weakness). All patients had to have an upright forced vital capacity (FVC) > 70%, to avoid overestimation of cognitive deficits due to respiratory muscle impairment.(18) Patients with ALS-bvFTD and bvFTD had possible or probable bvFTD, according to current criteria.(19) They could be sporadic or familial cases and were included irrespective of disease duration. Healthy controls had to have no history of neurological or psychiatric disease. All participants had to be older than 18 years, be fluent in Dutch and have a reliable proxy (partner, relative or close friend), also fluent in Dutch and willing to fill out questionnaires. We excluded

participants with other neurological or psychiatric conditions associated with cognitive or behavioural impairment, and participants who used high dose of psychotropic medication or more than 5 alcohol units per day.

3. Neuropsychological test battery

Order	Cognitive test	Cognitive domain	Items (N)	Parallel version
1	Dutch adult reading test (DART)	Verbal IQ	50	No
2	Benton temporal orientation test (BTOT)	Temporal orientation	5	No
3	Anti-saccade test	Executive functions	20	No
4	Similarities (subtest of WAIS-IV)	Language	19	No
5	Boston naming test (BNT)	Language	20	Yes
6	Judgment of line orientation (JOLO)	Visuospatial functions	30	No
7	Letter fluency index	Executive functions	n/a	Yes
8	Category fluency (animals, occupation, supermarket)	Executive functions	n/a	No
9	Visual association test	Visual memory	12 or 24	No
10	Rey auditory verbal learning test (RAVLT)	Verbal memory	7	Yes
11	Letter Number sequencing	Attention	10	No
12	Rivermead behavioral memory test (RBMT)	Verbal memory	2	Yes
13	Ekman 60 faces test	Social cognition	60	No
14	Wisconsin card sorting test (WCST)	Executive functions	n/a	No

Legend. The neuropsychological test protocol was always performed in this order. Responses to all tests, except the DART, Ekman 60 faces test and WCST, could be performed in writing, when dysarthria was severe. The Ekman 60 faces test and WCST were computerized, but

responses could be given orally, when hand function was severely impaired. The letter fluency index was used to correct for speech impairment; it consists of two conditions, the generation condition (the participant is asked to name as many words beginning with a certain letter in one minute, the produced words are written down by the examiner) and the control condition (the participant is asked to read aloud these produced words as quickly as possible). The fluency index is calculated as follows:
(time needed for generation - time needed for reading) / total number of items generated. The verbal fluency index reflects the average thinking time per word. For the visual association test, the neuropsychologist chose the appropriate version (short or long version), based on age.

Beeldman E, *et al. J Neurol Neurosurg Psychiatry* 2020; 91:779–780. doi: 10.1136/jnnp-2020-322992

4. Measures of motor function, respiratory function, anxiety and depression

The ALS functional rating scale-revised (ALSFRS-R) was administered to patients with ALS (score ranges from 0 to 48; a higher score indicates less impairment).(20) FVC in upright position was assessed in ALS, as a measure of respiratory muscle weakness (percentage of predicted value, corrected for age, sex and height). All participants filled out the hospital anxiety and depression scale (HADS; a higher score indicates more anxiety/depression, with a clinical threshold of 7 on subscales for anxiety and depression. Item 8 "I feel as if I'm slowed down" was removed, to avoid bias due to motor impairment.).(21)

5. Participant and disease characteristics at baseline

	**ALS**<br>**N = 35**	**BvFTD**<br>**N = 21**	**HC**<br>**N = 18**	**p-value**
Age (years)	63.8 (8.4)	64.6 (10.2)	60.9 (10.0)	0.4
Male (No.,%)	24 (68.6)	16 (76.2)	9 (50.0)	0.2
Education (years)	14 (6-18)	14 (10-18)	14.5 (10-18)	0.3
Disease duration (months)[1]	8 (4-15)	29 (10-168)	n/a	**<0.001**
Site of onset (l/b/lb)	18/15/2	n/a	n/a	n/a
ALSFRS-R score	42 (31-46)	n/a	n/a	n/a
FVC	94.0 (16.6)	n/a	n/a	n/a
Survival (months from symptom onset to death)[2]	32 (11-73)	n/a	n/a	n/a
C9orf72 mutation (No.,%)[3]	3 (10.3)	3 (23.1)	n/a	0.3
HADS anxiety	4 (0-13)	5 (0-9)	3 (0-7)	**<0.01**
HADS depression	1 (0-10)	3 (0-8)	0 (0-3)	0.4

Legend. Data are expressed as mean (SD) or median (range), where appropriate. ALS: amyotrophic lateral sclerosis; bvFTD: behavioural variant frontotemporal dementia; HC: healthy control; N: number; Site of onset (l/b/lb): limb onset/bulbar onset/limb and bulbar onset; ALSFRS-R: ALS functional rating scale-revised (range 0 to 48, higher score indicates less impairment); FVC: forced vital capacity, percentage of predicted value; n/a: not available; HADS: hospital anxiety and depression scale score, higher scores indicate more symptoms.[1] Disease duration was checked after inclusion and was longer than 12 months in 3 patients (8.6%). [2] By September 26, 2019 (censoring date) 32 patients with ALS (91.4%) and 4 patients with bvFTD (19.1%) had died. [3]*C9orf72* mutation status was missing in 6 patients with ALS and 8 patients with bvFTD.

Beeldman E, *et al. J Neurol Neurosurg Psychiatry* 2020; 91:779–780. doi: 10.1136/jnnp-2020-322992

6. Comparison of relevant participant and disease characteristics at baseline between patients with ALS with and without cognitive and/or behavioural impairment at baseline

	**No CI/BI**<br>**N = 19**	**Mild CI/BI**<br>**N = 9**	**Severe CI/BI**<br>**N = 7**	**p-value**
Site of onset (l/b/lb)	11/7/1	4/4/1	3/4/0	0.8
C9orf72 mutation (No.,%)[1]	1 17	0 6	2 6	0.1
HADS anxiety	4 (0-12)	3 (1-13)	5 (2-11)	0.1
HADS depression	1 (0-10)	3 (0-6)	5 (0-7)	0.5

Legend. Data are expressed as mean (SD) or median (range), when appropriate. CI/BI: cognitive and or behavioural impairment; N: number; Site of onset (l/b/lb): limb onset/bulbar onset/limb and bulbar onset; HADS: hospital anxiety and depression scale score, higher scores indicate more symptoms.[1] *C9orf72* mutation status was missing in 6 patients with ALS.

Beeldman E, *et al. J Neurol Neurosurg Psychiatry* 2020; 91:779–780. doi: 10.1136/jnnp-2020-322992

7. Change scores for neuropsychological tests and behavioural assessments at follow-up

Cognitive test		ALS N=28	BvFTD N=19	HC N=18	p-value
Anti-saccade test		-0.7 (2.9)	-1.1 (2.8)	-0.8 (2.1)	0.9
WAIS similarities		0.8 (3.1)	-0.6 (3.8)	0.9 (2.6)	0.4
Boston naming test		-0.9 (4.9)	-2.2 (5.5)	0.2 (1.7)	0.2
Judgement of line orientation		-0.5 (3.0)	0.0 (2.5)	-0.5 (2.2)	0.4
Letter fluency		-1.8 (5.7)*	0.9 (6.0)**	9.3 (6.4)	**<0.001**
Category fluency	Animals	-3.0 (5.5)	-1.5 (2.4)	-1.1 (3.7)	0.4
	Occupation	-2.6 (3.9)*	-0.5 (2.2)	-0.2 (3.2)	**0.04**
	Supermarket	-3.2 (5.4)*	-1.0 (4.0)	1.4 (5.3)	**0.02**
Visual association test		-0.9 (4.1)	0.0 (3.6)	-0.5 (5.7)	0.5
RAVLT	Immediate	-0.1 (7.4)	-1.8 (8.6)	4.5 (8.0)	0.2
	Delayed	-0.1 (2.1)*	-0.1 (3.1)**	2.1 (2.65)	**0.02**
Letter number sequencing		0.2 (1.9)	-1.4 (3.3)	0.7 (2.4)	0.1
RBMT	Immediate	0.8 (5.0)	-1.0 (4.3)	1.9 (4.6)	0.1
	Delayed	0.4 (5.1)	-1.1 (4.0)	1.4 (4.2)	0.3
Ekman 60 faces test		-0.9 (3.6)*	-4.4 (6.7)**	2.3 (4.3)	**<0.01**
WCST	Total errors	3.0 (15.4)	15.3 (25.6)	2.4 (6.9)	0.1
	Perseverative responses	0.9 (15.3)	13.8 (23.6)**	1.2 (4.2)	**0.04**
	Perseverative errors	1.4 (12.1)	11.5 (18.7)	1.0 (2.6)	0.1
ALS-FTD-Q[#]		-2.7 (10.3)	-1.4 (7.9)	2.5 (3.6)	0.1

Legend. ALS: amyotrophic lateral sclerosis; bvFTD: behavioural variant frontotemporal dementia; HC: healthy controls; RAVLT: Rey auditory verbal learning test; RBMT: Rivermead behavioural memory test; WCST: Wisconsin card sorting test; ALS-FTD-Q: ALS-

Beeldman E, *et al. J Neurol Neurosurg Psychiatry* 2020; 91:779–780. doi: 10.1136/jnnp-2020-322992

FTD questionnaire. Change scores for all participants with follow up (ALS 28/35, HC 18/18, bvFTD 19/21). Differences between all groups are calculated with the Kruskal-Wallis test, and when significant, with the Mann-Whitney U test for ALS vs. HC (*p < 0.05, *p < 0.001) and FTD vs. HC (**p < 0.05, **p <0.001). #Change scores of the ALS-FTD-Q did not differ between all groups (ALS, bvFTD and HC); however, when ALS and HC were compared, a p-value of $p = 0.04$ was found.

Beeldman E, *et al. J Neurol Neurosurg Psychiatry* 2020; 91:779–780. doi: 10.1136/jnnp-2020-322992

8. Comparison of change scores of motor function, respiratory functions and anxiety and depression between patients with ALS with and without category shifts

Between patients with (n=10) and without (n=18) a shift towards a more severe category of cognitive or behavioural impairment, change scores of ALSFRS-R (-4.0 (range -18.0 – 1.0) vs. -6.5 (range -23.0 – 0.0)), FVC (-11.2 (range -50.2 – 24.7) vs. -16.9 (range -45.8 – 10.0)) and HADS (-2.0 (range -6.0 – 1.0) vs. -0.5 (range -6.0 – 4.0)) did not differ significantly (p = 0.2, 0.4 and 0.4, respectively).

Beeldman E, *et al. J Neurol Neurosurg Psychiatry* 2020; 91:779–780. doi: 10.1136/jnnp-2020-322992

9. References

1. Burkhardt C, Neuwirth C, Weber M. Longitudinal assessment of the Edinburgh Cognitive and Behavioural Amyotrophic Lateral Sclerosis Screen (ECAS): lack of practice effect in ALS patients? Amyotroph Lateral Scler Frontotemporal Degener. 2017;18(3-4):202-9.
2. Floeter MK, Traynor BJ, Farren J, Braun LE, Tierney M, Wiggs EA, et al. Disease progression in C9orf72 mutation carriers. Neurology. 2017;89(3):234-41.
3. Poletti B, Solca F, Carelli L, Faini A, Madotto F, Lafronza A, et al. Cognitive-behavioral longitudinal assessment in ALS: the Italian Edinburgh Cognitive and Behavioral ALS screen (ECAS). Amyotroph Lateral Scler Frontotemporal Degener. 2018;19(5-6):387-95.
4. Woolley S, Goetz R, Factor-Litvak P, Murphy J, Hupf J, Lomen-Hoerth C, et al. Longitudinal Screening Detects Cognitive Stability and Behavioral Deterioration in ALS Patients. Behav Neurol. 2018;2018:5969137.
5. Xu Z, Alruwaili ARS, Henderson RD, McCombe PA. Screening for cognitive and behavioural impairment in amyotrophic lateral sclerosis: Frequency of abnormality and effect on survival. J Neurol Sci. 2017;376:16-23.
6. Abrahams S, Leigh PN, Goldstein LH. Cognitive change in ALS: a prospective study. Neurology. 2005;64(7):1222-6.
7. Elamin M, Bede P, Byrne S, Jordan N, Gallagher L, Wynne B, et al. Cognitive changes predict functional decline in ALS: a population-based longitudinal study. Neurology. 2013;80(17):1590-7.
8. Gillingham SM, Yunusova Y, Ganda A, Rogaeva E, Black SE, Stuss DT, et al. Assessing cognitive functioning in ALS: A focus on frontal lobe processes. Amyotroph Lateral Scler Frontotemporal Degener. 2017;18(3-4):182-92.
9. Kasper E, Zydatiss K, Schuster C, Machts J, Bittner D, Kaufmann J, et al. No Change in Executive Performance in ALS Patients: A Longitudinal Neuropsychological Study. Neuro-degenerative diseases. 2016;16(3-4):184-91.
10. Kilani M, Micallef J, Soubrouillard C, Rey-Lardiller D, Demattei C, Dib M, et al. A longitudinal study of the evolution of cognitive function and affective state in patients with amyotrophic lateral sclerosis. Amyotroph Lateral Scler Other Motor Neuron Disord. 2004;5(1):46-54.
11. Proudfoot M, Menke RA, Sharma R, Berna CM, Hicks SL, Kennard C, et al. Eye-tracking in amyotrophic lateral sclerosis: A longitudinal study of saccadic and cognitive tasks. Amyotroph Lateral Scler Frontotemporal Degener. 2015;17(1-2):101-11.
12. Robinson KM, Lacey SC, Grugan P, Glosser G, Grossman M, McCluskey LF. Cognitive functioning in sporadic amyotrophic lateral sclerosis: a six month longitudinal study. Journal of neurology, neurosurgery, and psychiatry. 2006;77(5):668-70.
13. Schreiber H, Gaigalat T, Wiedemuth-Catrinescu U, Graf M, Uttner I, Muche R, et al. Cognitive function in bulbar- and spinal-onset amyotrophic lateral sclerosis. A longitudinal study in 52 patients. Journal of neurology. 2005;252(7):772-81.
14. Strong MJ, Grace GM, Orange JB, Leeper HA, Menon RS, Aere C. A prospective study of cognitive impairment in ALS. Neurology. 1999;53(8):1665-70.
15. De Silva D, Hsieh S, Caga J, Leslie FV, Kiernan MC, Hodges JR, et al. Motor function and behaviour across the ALS-FTD spectrum. Acta Neurol Scand. 2016;133(5):367-72.
16. Brooks BR, Miller RG, Swash M, Munsat TL. El Escorial revisited: revised criteria for the diagnosis of amyotrophic lateral sclerosis. Amyotroph Lateral Scler Other Motor Neuron Disord. 2000;1(5):293-9.

17. van der Graaff MM, Sage CA, Caan MW, Akkerman EM, Lavini C, Majoie CB, et al. Upper and extra-motoneuron involvement in early motoneuron disease: a diffusion tensor imaging study. Brain. 2011;134(4):1211-28.
18. Newsom-Davis IC, Lyall RA, Leigh PN, Moxham J, Goldstein LH. The effect of non-invasive positive pressure ventilation (NIPPV) on cognitive function in amyotrophic lateral sclerosis (ALS): a prospective study. Journal of neurology, neurosurgery, and psychiatry. 2001;71(4):482-7.
19. Rascovsky K, Hodges JR, Knopman D, Mendez MF, Kramer JH, Neuhaus J, et al. Sensitivity of revised diagnostic criteria for the behavioural variant of frontotemporal dementia. Brain. 2011;134(Pt 9):2456-77.
20. Cedarbaum JM, Stambler N, Malta E, Fuller C, Hilt D, Thurmond B, et al. The ALSFRS-R: a revised ALS functional rating scale that incorporates assessments of respiratory function. BDNF ALS Study Group (Phase III). J Neurol Sci. 1999;169(1-2):13-21.
21. Spinhoven P, Ormel J, Sloekers PP, Kempen GI, Speckens AE, Van Hemert AM. A validation study of the Hospital Anxiety and Depression Scale (HADS) in different groups of Dutch subjects. Psychol Med. 1997;27(2):363-70.
